# An International Perspective on the Impacts of COVID-19 on Adult Education and Training

**DOI:** 10.1093/geroni/igab046.563

**Published:** 2021-12-17

**Authors:** Oksana Dikhtyar, Abigail Helsinger, Phyllis Cummins, Nytasia Hicks

**Affiliations:** 1 Scripps Gerontology Center, Miami University, Oxford, Ohio, United States; 2 Miami University, Oxford, Ohio, United States; 3 GRECC - STVHCS Department of Veterans Affairs, San Antonio, Texas, United States

## Abstract

The COVID-19 pandemic has caused one of the worst economic crises since the Great Depression and the current recession has been more detrimental to older workers compared to other age groups. Not only has it forced more older workers out of their jobs, but it has also made it much harder for jobless older workers to find a new job. Furthermore, due to increased automation and digitalization in the workplace, older workers will likely need upskilling or reskilling to improve their employment prospects in the changed labor market. This situation brings the importance of offering training and continuous education programs that target older workers to the forefront of adult education policy and practice. This qualitative study examines measures taken in response to COVID-19 in adult education and training (AET) in seven countries including Sweden, Norway, the Netherlands, Australia, Singapore, Canada, and the United States. The findings are based on key informant interviews with international policy experts and scholars in the field of AET in addition to information gathered from written materials (e.g., government and organizational reports). To expedite their economic recovery and improve labor market outcomes for their workers, some countries have increased government funding for vocational and continuing education or offered financial support for post-secondary students while others have provided funds to employers to offer training and retraining for their employees. Some of these measures have the potential to expand adult educational opportunities in the post-pandemic world. Implications for policy and practiced are discussed.

